# Altered Body Composition of Psoas and Thigh Muscles in Relation to Frailty and Severity of Parkinson’s Disease

**DOI:** 10.3390/ijerph16193667

**Published:** 2019-09-29

**Authors:** Cheng-Kang Wang, Hsiu-Ling Chen, Cheng-Hsien Lu, Meng-Hsiang Chen, Pi-Ling Chiang, Yueh-Sheng Chen, Wei-Che Lin

**Affiliations:** 1 Department of Diagnostic Radiology, Kaohsiung Chang Gung Memorial Hospital, Chang Gung University College of Medicine, 123 Ta-Pei Road, Niao-Sung, Kaohsiung 83305, Taiwan; 2 Department of Neurology, Kaohsiung Chang Gung Memorial Hospital, Chang Gung University College of Medicine, 123 Ta-Pei Road, Niao-Sung, Kaohsiung 83305, Taiwan

**Keywords:** body composition, frailty, sarcopenia, Parkinson disease, magnetic resonance imaging

## Abstract

**Background**: To investigate the relationship between fat content and the cross-sectional area of psoas and thigh muscles, and clinical severity in patients with Parkinson’s disease. **Materials and Methods**: Twenty-five patients and 20 age- and sex-matched normal controls were recruited. All subjects underwent MRI study to determine the fat content of the bilateral psoas and thigh muscles. Muscle quality was measured by grasp, walking speed, and cross-sectional area. All patients underwent clinical surveys to evaluate disease severity and frailty, and analyses of the correlations between muscle quality and disease severity were performed. **Results**: Compared with the controls, patients exhibited higher fatty content in the measured muscles. The higher fat infiltration of measured muscles was significantly correlated with increased disease severity and frailty in patients. The fat fraction of the bilateral medial compartment of the thigh was correlated with the Unified Parkinson Disease Rating Scale-I results and the fat fraction of the bilateral anterior compartment of the thigh was correlated with weakness and exhaustion in patients. **Conclusions**: Decreased quality in psoas and thigh muscles is prominent in Parkinson’s disease which is further associated with disease severity and frailty. Awareness of the risk of sarcopenia and associated sequelae might improve patient care and outcomes.

## 1. Introduction

Parkinson’s disease (PD) is a neurodegenerative disorder associated with loss of muscle strength [1]. The lesion-side was the weaker side as compared with the other side in movement [1]. PD-related weakness has also been linked to the duration and severity of PD [2]. It can be surmised that the integrity of the musculoskeletal system should be affected as the disease progresses; however, research focusing on detailed intramuscular component analysis and the relationship between muscle content and neuronal degeneration and clinical severity has been limited [3].

With disease progression, muscle weakness may also be associated with frailty in PD patients [4]. Frailty has been defined as a syndrome consisting of the presence of five phenotypic criteria, including low grip strength, unintentional weight loss, a slower walking speed, self-reported exhaustion, and low physical activity [5]. Frailty is highly concomitant with sarcopenia exhibited as the age-related loss of muscle mass, strength, and function. Sarcopenia can result not only in an impaired ability to perform activities but also in increased mortality in elders [6]. PD and sarcopenia are both age-related syndromes and the motor symptoms of PD can be associated with early-stage sarcopenia [7]. It is possible that both PD and aging share a common pathway in terms of the changes to body composition and frailty; however, clinicians have little information about the relationships among the frailty, severity of PD, and changes in body composition.

Numerous techniques can provide information about body composition analysis. Dual-energy X-ray absorptiometry (DXA) is the most common measurement of body composition. It can provide noninvasive estimates of bone mineral, fat-free mass, and fat mass [8]. The advantages of DXA are its good accuracy and reproducibility. However, the use of DXA entails radiation exposure. Magnetic resonance imaging (MRI) has been well established to separate fat and water proton signals and have been applied for the classification of fat distribution into subcutaneous, visceral, and intramuscular fat [9]. The iterative decomposition of water and fat with echo asymmetry and least-squares estimation (IDEAL) technique [10], which reconstruct fat-only and water-only images might be useful for evaluations of intramuscular component alterations, which might serve, in turn, as a new biomarker for early sarcopenia detection. 

The aims of this study were to investigate the levels of fat content and cross-sectional area of psoas and thigh muscles in PD groups and normal control (NC) groups and to correlate the measured components with frailty and disease severity in the PD groups by using the IDEAL technique and frailty assessments.

## 2. Materials and Methods

The hospital’s Institutional Review Committee on Human Research approved the study protocol, and all of the participants or their guardians provided written informed consent.

### 2.1. Subjects

Twenty-five patients with PD (5 men and 20 women; mean age: 63.60 ± 5.54 years; range: 56–71 years old) were recruited. All the PD patients were diagnosed by experienced neurologists according to the Parkinson’s Disease Society’s criteria [11], and their disease severity and functional status were evaluated by the Unified Parkinson Disease Rating Scale (UPDRS) [12], modified Hoehn and Yahr staging (H&Y) scale [13], and the Schwab and England Activities of Daily Living (S&E ADL) Scale [14] in the “OFF” state. 

Twenty age- and sex-matched healthy subjects (4 men and 16 women; mean age: 63.00 ± 4.09 years; range: 59–72 years old) without alcohol abuse, neurologic disease, or psychiatric illness, and with similar education levels were recruited from the hospital as a control group. The hospital’s Institutional Review Committee on Human Research approved the study protocol and all of the participants or their guardians provided written informed consent.

### 2.2. Assessment of Disease Severity, Frailty and Muscle Quality

The clinician assessed the PD patients with the UPDRS via clinical observation and interview regarding multiple aspects of PD, including mental dysfunction and four parts of the UPDRS. The modified H&Y scale was used to evaluate the severity of PD according to clinical findings and functional disability. The S&E ADL Scale was used to assess relative independence.

We used the modified Cardiovascular Health Study Phenotypic Classification of Frailty (CHS_PCF) and the Taiwan International Physical Activity Questionnaire Short Form (IPAQ-SF) to evaluate the study participants in terms of the five components of frailty [15] because it has been validated in Taiwanese populations. These components of frailty included unintentional weight loss, low energy and exhaustion, low physical activity, slowed walking speed, and low grip strength. A hand dynamometer was used to assess the average grip strength values of the bilateral hands over three measurements, with the threshold value for low grip strength being based on BMI and gender. The definition of frail was met if the patient had scores on the lesion-side of the threshold values for more than three components. The muscle quality is typically defined as muscle strength [16], which was measured by the walking speed and grip strength as the frailty assessment in this study.

### 2.3. Magnetic Resonance Imaging (MRI) Data Acquisition

Magnetic resonance scanning was performed on a 1.5T MRI system (Discovery 450, GE Healthcare, Milwaukee, Wis, United States). A body coil was used for signal excitation and a 12-channel body phased-array coil was used for signal reception. 

### 2.4. IDEAL

The IDEAL sequence (IDEAL IQ, GE Healthcare) was employed for the evaluation of the percentages of fat content in the bilateral psoas muscles and the three compartments of each thigh. The IDEAL IQ technique is a T1-independent, T2*-corrected chemical shift-based fat-water separation method with multipeak fat spectral modeling. The imaging parameters for the IDEAL IQ technique were as follows: flip angle = 5; echo time = 1.3, 3.3, 5.3, 7.3, 9.3, and 11.3 milliseconds; repetition time = 13.7 milliseconds; bandwidth = 61.25 kHz; field of view = 40 × 40 cm at psoas muscle region and 42 × 42 cm at thigh region; slice thickness = 10 mm; matrix size = 256 × 128; number of slices = 32 at psoas muscle region and 36 at the thigh region. The IDEAL IQ technique produces fat, water, in-phase, and out-phase function maps as well as T2*-corrected water and, T2*-corrected fat, fraction maps. 

### 2.5. Regions of Interest (ROI)

**Figure 1:**
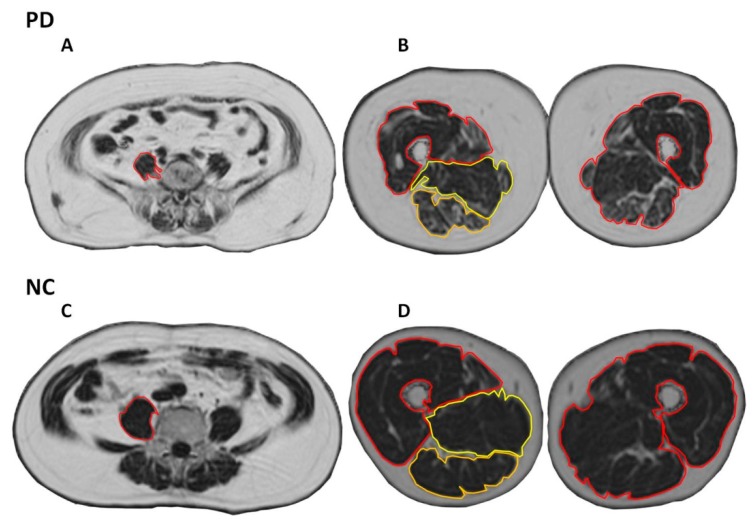
The regions of interest (ROI) of IDEAL fat fraction map images. The ROI included bilateral psoas muscles, and three compartments and the total of bilateral thighs. **A** and **C**: Psoas muscles. **B** and **D**: Three compartments of thigh. Abbreviations: NC: normal control; PD: Parkinson’s disease.

To estimate the fat fraction of the bilateral psoas muscles and the three compartments of each thigh, the signal intensity from the regions of interest (ROI) of those areas was calculated in an IDEAL fat fraction map image (Figure 1). The signal intensity in an IDEAL fat fraction map image is approximately equal to the fat fraction in muscles. All measurements were performed by two radiologists (LWC and WCK), with agreement required to ensure the appropriate positions of the ROIs. The measured levels of the psoas muscles and the thighs were at the L5 vertebral body and at the femoral shaft, which is 20 cm from the femoral head, respectively. The measured ROI areas included the psoas muscles, and the anterior, medial, and posterior compartment of each thigh.

### 2.6. Assessment of Cross-Sectional Area

The compartment cross-sectional areas (mm^3^) were measured according to the ROI of the thigh compartments in an IDEAL fat fraction map image. All measurements were also performed by two radiologists (LWC and WCK) with agreement required to ensure the appropriate areas of the ROIs. 

### 2.7. Statistical Analysis 

#### 2.7.1. Comparison Between Normal Controls and PD Patients 

SPSS software (SPSS V.22, Chicago, IL, USA) was used to perform all the statistical analyses. The demographic data, including the age and sex data, were compared with the study groups using the 2-sample Student t-test and Pearson chi-square test and were reported as mean ± the standard deviation (SD). 

The significance of differences in disease severity, the components of frailty, fat content, and muscle area were analyzed by analysis of covariance (ANCOVA) with the participant’s age, sex, and body mass index (BMI) as covariates. The threshold for statistical significance was set at *p* < 0.05.

#### 2.7.2. Correlations

Because the characteristics of weight loss, weakness, exhaustion, and overall frailty status are nominal scales and the characteristics of physical activity, slowness, and grip strength are interval scales, we normalized those characteristics to standardized z-score values for further comparison. Partial correlation analysis was performed with age, sex, and BMI adjustments to determine the associations among the percentage of fat content and area of muscles, disease severity, and the z-scores of frailty. The threshold for statistical significance was set at *p* < 0.05. 

## 3. Results

### 3.1. Demographic Characteristics of the Participants: 

The demographic characteristics, clinical severity, and frailty evaluation results of the PD patients and NC are shown in Table 1. The normal controls and PD patients had similar mean ages and sex distributions (age: *p* = 0.688; sex: *p* = 1.000) and there were no significant group differences in body height, bodyweight, or BMI (height: *p* = 0.951; body weight: *p* = 0.182; BMI: *p* = 0.091). The mean disease duration of PD patients was 1.70 ± 2.15 years and their mean Levodopa equivalent dose was 482.58 ± 359.98 mg/day. The mean H&Y stage of the PD patients was 2.02 ± 1.08, indicating mild to early moderate disease severity. The distributions of the UPDRS I, UPDRS II, UPDRS III, UPDRS total, and S&E scale results are all listed in Table 1.

### 3.2. Frailty

Significant difference between the two groups were found for all the components of the frailty evaluation, including bodyweight loss (PD: n = 8, 32%; NC: n = 1, 5%; *p* = 0.048), weakness (PD: n = 18, 72%; NC: n = 2, 10%, *p* < 0.001), exhaustion (PD: n = 18, 72%; NC: n = 2, 10%; *p* < 0.001), walking time (PD: n = 12, 48%; NC: n = 0, 0%; *p* = 0.048), and grip strength of the right hand (PD: n = 11, 44%; NC: n = 3, 15%; *p* = 0.029) and left hand (PD: n = 14, 56%; NC: n = 3, 15%; *p* < 0.001), with the exception of physical activity (PD: n = 12, 48%; NC: n = 9, 45%; *p* = 0.081).

### 3.3. IDEAL

#### 3.3.1. The Percentage of Fat Content

**Figure 2:**
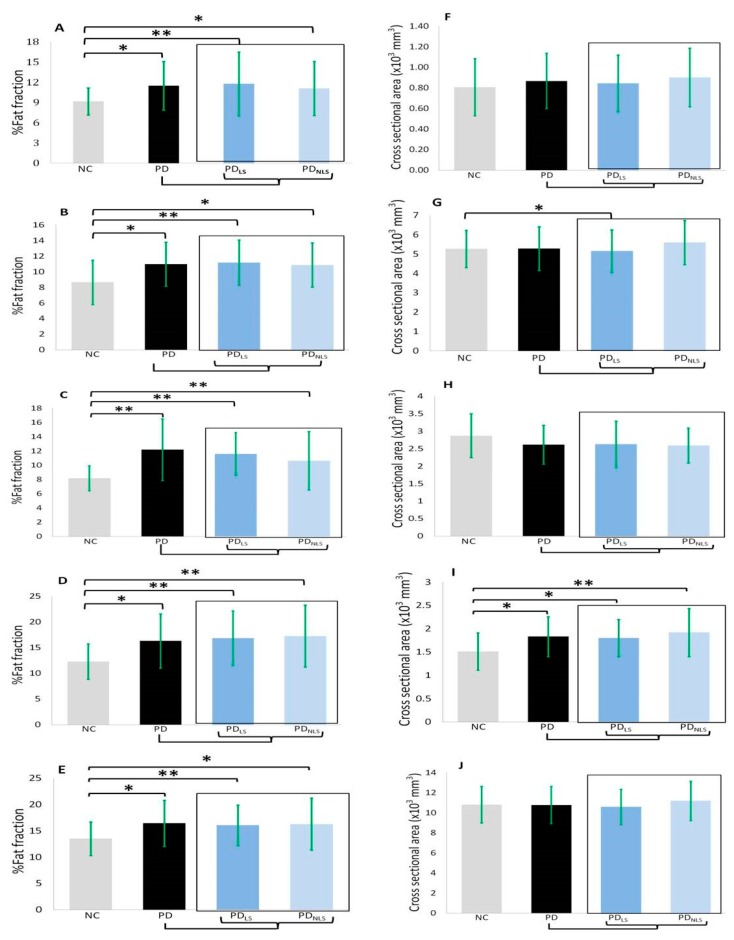
Fat fraction and cross-sectional areas of five ROI among NC, PD, lesion and non-lesion side of PD. **A** and **F**: psoas muscle; **B** and **G**: anterior compartment of thigh; **C** and **H**: medial compartment of thigh; **D** and **I**: posterior compartment of thigh; **E** and **J**: total compartment of thigh. Abbreviations: NC: normal control; PD: Parkinson’s disease; LS: lesion side; NLS: non-lesion side. Fat fraction and cross-sectional data were compared by analysis of covariance (ANCOVA) after controlling for age, sex and BMI. *: *p* < 0.05; **: *p* < 0.01.

The body composition data of the participants are shown in Table 2, Table 3, and Figure 2. The PD patients displayed higher percentages of fat content in terms of the mean values for the bilateral psoas muscles (*p* = 0.011) and the three compartments of the bilateral thighs compared to the NC (anterior compartment: *p* = 0.0019; medial compartment: *p* = 0.001; posterior compartment: *p* = 0.010; total thigh: *p* = 0.019). Both the lesion side and non-lesion side of the PD patients had significantly higher percentages of fat content when compared to the corresponding regions in the NC (lesion side: psoas muscle: *p* = 0.003; anterior compartment: *p* = 0.002; medial compartment: *p* < 0.001; posterior compartment: *p* < 0.001; total thigh: *p* = 0.008; non-lesion side: psoas muscle: *p* = 0.038; anterior compartment: *p* = 0.016; medial compartment: *p* = 0.007; posterior compartment: *p* < 0.001; total thigh: *p* = 0.026). However, there was no significant difference in the percentages of fat content between the lesion side and non-lesion side within the PD patients group itself (psoas muscle: *p* = 0.518; anterior compartment: *p* = 0.540; medial compartment: *p* = 0.560; posterior compartment: *p* = 0.735; total compartment: *p* = 0.853). 

#### 3.3.2. Cross-Sectional Areas

The compartment cross-sectional areas (mm^3^) were also compared between the two groups (Table 2, Table 3, and Figure 2). Two compartments of the thigh exhibited a significantly lower cross-sectional area in the lesion side of the PD patients compared with the corresponding areas in the NC (anterior compartment: *p* = 0.027; medial compartment: *p* = 0.032; total thigh: *p* = 0.004) after controlling for sex, age, and BMI. The posterior compartment of the thigh in the PD group showed higher cross-sectional area compared with the corresponding areas in the NC but there was no significant difference between the two groups. There was no significant difference between the lesion side and non-lesion side within the PD patient group itself.

### 3.4. Analysis in Gender Groups

Body composition differs between men and women, more specifically, women have higher body fat and less muscle mass than men. The subgroup analysis of gender differences between the PD and NC groups is shown in Table 4. In the fat fraction, the female PD group showed higher fat content than the female NC group in the psoas and thigh compartments. Meanwhile, no significant differences were noted between the male PD male and NC groups.

### 3.5. Correlations

#### 3.5.1. Correlations among the Percentage of Fat Content, Disease Severity, and Frailty

**Figure 3:**
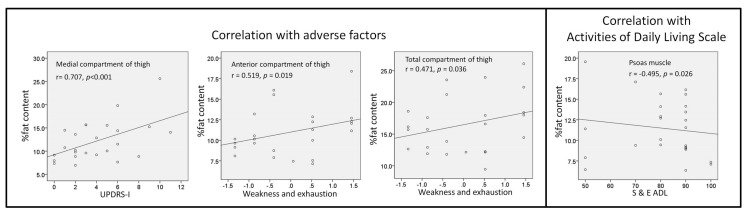
Among the percentage of fat content, disease severity, and frailty. The Unified Parkinson’s Disease Rating Scale (UPDRS) is a comprehensive 50 question assessment of both motor and non-motor symptoms associated with Parkinson’s disease. Weakness and exhaustion are parts of frailty, including lower grip strength and self-reported exhaustion, identified by two questions from the CES–D scale. Schwab and England Activities of Daily Living Scale is an assessment of an individual’s ability to function in activities of daily living. Rated in 10% increments: 1) 100% = completely independent, 2) 0% = vegetative.

Higher percentages of fat content in the medial compartment of the thigh were associated with higher UPDRS-I scores (r = 0.707, *p* < 0.001), as showed in Figure 3. Higher weakness and exhaustion scores were correlated with higher percentages of fat content in anterior compartment of the thigh and total thigh (r = 0.519, *p* = 0.019 and r = 0.471, *p* = 0.036).

#### 3.5.2. Correlations among Compartment Area, Disease Severity, and Frailty

There were no significant associations between the cross-sectional areas and the clinical evaluation parameters in the PD patients.

## 4. Discussion

After adjusting for BMI, we found that the PD patients presented poor muscle integrity, which was muscle components and performance, such as slow walking speed, lower grip strength, and the elevated degree of fatty replacement that occurred in the core muscles and bilateral thighs. Increased fatty content and decreased lean mass, indicating sarcopenia, were highly associated with disease severity. Furthermore, poor muscle integrity was also correlated with certain frailty evaluation results, such as the weakness and exhaustion scores. In Parkinson’s disease, poor muscle integrity and sarcopenia may be the common downstream pathway. The relationship between PD and sarcopenia might lead to a vicious cycle of further frailty and disease deterioration in PD. 

PD is considered to be a disease resulting in disrupted brain networks and motor function impairment. The major contributors to frailty and motor impairment are recognized as balance and gait disturbances [17]. Several central and peripheral mechanisms of those symptoms have been proposed [17]. The major pathophysiology of bradykinesia and tremor is thought to be a central origin. Failure of the basal ganglia output seems to be an important central factor [18]. In terms of peripheral origin, abnormalities in the passive mechanical properties and peripheral sensory inputs may influence muscle movement [18]. In a previous study, it was confirmed that action tremor contributes to muscle weakness [19]. Therefore, the main symptoms of PD might be associated with frailty and changes in the quality of the muscles. Sarcopenia is also considered to be an aspect of frailty, such that its defining criteria and pathophysiology overlap to some extent with those of frailty. Both of the two conditions are often comorbid and present with the same phenotype, including weakness and slowness, due to the associated loss of muscle mass and strength [20]. Sarcopenia is believed to be a precursor syndrome to frailty and decreased lower-limb muscle strength has previously been found to be associated with a high risk of frailty [21]. In general, neurodegenerative disorders present clinically with motor impairments and are commonly associated with frailty and sarcopenia [7,22]. 

The MRI IDEAL technique provides detailed data regarding the fat content of each muscle group. Therefore, MRI is considered the gold standard for measurements of muscle mass and size [23]. By contrast, the poor spatial discrimination ability of DXA might cause it to underestimate individual muscle sizes and the degrees of sarcopenia [23]. Computed tomography (CT) can distinguish visceral from subcutaneous fat [24]; however, it involves even higher levels of radiation exposure than DXA does. The IDEAL sequence of MRI can separate fat and water with an optimal signal-to-noise ratio (SNR) allowing for a wider array of applications, including T1 weighted image (T1WI), T2 weighted image (T2WI), and proton density imaging. Compared with IDEAL sequence, Short-T1 inversion recovery (STIR) imaging shows uniform fat suppression but with lower SNR, thus limiting STIR imaging to T2WI applications [25,26]. Meanwhile, although both CT and MRI scans can provide imaging of fatty infiltrations and cross-sectional areas of given muscles [27], CT presents poor soft tissue resolution quality compared with MRI, with the added consideration of radiation exposure. In our study, MRI showed higher fat content in the PD group than in the NC group. The loss of muscle mass and the increased fat content in the PD patients were likely due in large part to their decreased motor function, which has been reported to affect both the activity and lifestyle of those with PD. Our further analysis demonstrated that the changes of higher intramuscular fat content in the PD patients were not accompanied with obvious changes in the cross-sectional area within a specific given region. This quality-first phenomenon in PD was consistent with the results of a previous study of sarcopenia due to aging [28].

Frailty and sarcopenia have substantial impacts on PD patients [4,29]. Consistent with our findings, thigh muscle mass has previously been found to represent whole-body muscle more accurate and to show a strong correlation with frailty [30]. The MRI IDEAL technique is a more precise and early detection method requiring no radiation exposure. A comparison with a previous study on body composition in PD measured by DXA [31] demonstrated that the IDEAL technique can separate fat and water precisely. Our study also showed that changes in body composition were correlated with disease severity and frailty. In clinical applications, frailty or sarcopenia may be misinterpreted as a functional deterioration in PD. However, this study demonstrated that the early detection of changes in body composition in PD is possible and such early detection would allow for early intervention with regular exercise, which is considered the only effective strategy against sarcopenia [32]. Sarcopenia can be observed from the earliest clinical stages of PD and further emerges when PD-associated neuropathology in the muscle has advanced sufficiently to increase weakness and frailty. Therefore, it is particularly important for clinicians to be aware of sarcopenia when conducting regular rehabilitation and training in early PD [33,34] in order to prevent muscle loss and its consequences.

In the subgroup analysis of gender, the female PD group showed significantly higher fat infiltrations in the psoas and thigh muscles than the female NC group. However, there was no significant difference between the male PD group and NC groups. As previously reported, women exhibit higher body fat and less muscle mass than men [35]. In female PD patients, the increased fat content may be attributed to both sex-based differences and disease-related factors.

MRI can precisely estimate lean mass change but it is difficult to apply it to a large sample of subjects. As such, our results might not be representative of all PD populations. Second, we observed higher fat infiltration in the measured muscles within the PD patients but the specific causes and effects of neurodegeneration and changes in body composition should be further clarified by further prospective studies in order to exclude confounders relating to lifestyle. Third, this study was a cross-sectional study, such that caution is warranted in the interpretation of its results. The causal relationships among changes in PD, body composition, and frailty need to be clarified by future longitudinal studies.

## 5. Conclusions

Higher fat content in psoas and thigh muscles is commonly observed in PD patients. The present study reveals the association between fat infiltrations and frailty, indicating that the pathophysiology of PD and frailty, and possibly sarcopenia, may be a consequentially related process involving specific pathways and factors. As MRI can provide imaging of precise changes to the body composition of PD patients and the elderly, the imaging data may be used to inform physicians and caregivers, who could subsequently introduce early intervention to prevent injury resulting from inadvisable daily activities.

## Figures and Tables

**Table 1:** Demographic data, disease severity, and assessments of frailty in patients with PD and control subjects.

***Clinical Demographics***	PD (n = 25)	Control (n = 20)	*p*
Age (year)	63.60 ± 5.54	63.00 ± 4.09	0.688
Sex			
Male	5	4	
Female	20	16	
UPDRS I	4.04 ± 3.12		
UPDRS II	10.68 ± 5.41		
UPDRS III	27.92 ± 14.17		
UPDRS total	42.64 ± 20.24		
Modified H & Y (maximum stage is 5)	2.02 ± 1.08		
S & E (minimum points is 0 suggesting vegetative functions)	80.40 ± 15.41		
Disease duration (years)	1.70 ± 2.15		
Levodopa equivalent dose (mg/day)	482.58 ± 359.98		
Height (cm)	157.31 ± 5.44	157.20 ± 6.18	0.951
Body weight (kg)	62.06 ± 9.93	57.77 ± 11.25	0.182
Body Mass Index (BMI)	25.09 ± 3.80	23.14 ± 3.51	0.091
***Assessments of Frailty***			
Body weight loss (0: no; 1: yes)	0.42 ± 0.58	1.23 ± 0.44	0.048 *
Weakness	1.46 ± 1.14	0.15 ± 0.38	0.000 *
Exhaustion	1.42 ± 1.10	0.15 ± 0.38	0.000 *
Physical activity (× 10^2^ kcals/week)	2.17 ± 0.93	3.03 ± 1.91	0.081
Slowness (five-meter walking time; second)	7.96 ± 4.45	5.36 ± 0.58	0.048 *
Grip strength of Right hand (kg)	18.32 ± 7.32	22.85 ± 7.04	0.029 *
Grip strength of Left hand (kg)	15.73 ± 4.99	22.72 ± 7.42	0.000 *

Sex data were compared by Pearson chi-square test. Age data were compared by independent t test. Height, body weight, BMI and assessments of frailty data were compared by analysis of covariance (ANCOVA) after controlling for age and sex. Data are presented as mean ± standard deviation. * *p* < 0.05. Abbreviations: UPDRS, Unified Parkinson’s Disease Rating Scale; Modified H & Y, Modified Hoehn and Yahr stages; S & E, Schwab and England activities of daily living scale; kcal: kilocalories.

**Table 2:** A fat fraction and cross-sectional areas (mm^3^) of bilateral psoas muscles and thigh muscles in PD patients and normal controls.

		Patients with Parkinson’s Disease			
**Fat Fraction (%)**	Normal Controls	PD	PD_LS_	PD_NLS_	*p*
Psoas muscles	9.16 ± 2.01 ^§#^	11.49 ± 3.59	11.77 ± 4.71 ^§^	11.08 ± 4.01 ^#^	0.011 *
Anterior compartment of thigh	8.65 ± 2.83 ^§#^	10.97 ± 2.81	11.17 ± 2.91 ^§^	10.87 ± 2.84 ^#^	0.019 *
Medial compartment of thigh	8.18 ± 1.77 ^§#^	12.19 ± 4.34	11.59 ± 2.99 ^§^	10.62 ± 4.10 ^#^	0.001 *
Posterior compartment of thigh	12.27 ± 3.46 ^§#^	16.30 ± 5.28	16.83 ± 5.31 ^§^	17.26 ± 6.05 ^#^	0.010 *
Total thigh	13.51 ± 3.19 ^§#^	16.45 ± 4.37	16.05 ± 3.84 ^§^	16.27 ± 4.93 ^#^	0.019 *
**Cross-Sectional Area (mm^3^)**					
Psoas muscle	806.80 ± 276.63	867.38 ± 269.67	844.49 ± 275.11	901.71 ± 284.93	0.672
Anterior compartment of thigh	5267.33 ± 960.91 ^§^	5288.28 ± 1129.28	5152.86 ± 1094.41 ^§^	5601.82 ± 1140.72	0.246
Medial compartment of thigh	2872.52 ± 626.79 ^§#^	2615.18 ± 556.09	2628.61 ± 660.64^§^	2590.92 ± 501.83 ^#^	0.062
Posterior compartment of thigh	1511.22 ± 399.79 ^§#^	1832.25 ± 426.36	1800.75 ± 394.60 ^§^	1919.07 ± 513.87^#^	0.061
Total thigh	10,809.89 ± 1814.65 ^§^	10,781.20 ± 1843.18	10,590.24 ± 1771.51 ^§^	11,200.29 ± 1956.08	0.076

Fat fraction and cross-sectional data were compared by analysis of covariance (ANCOVA) after controlling for age, sex and BMI. The *p*-value represents the comparison the average of bilateral measured muscles between all PD patients v.s. the normal control group; data are presented as mean ± standard deviation; * *p* < 0.05 §: Significant differences between the normal group and the lesion side of the PD group by ANCOVA after controlling for age, sex, and BMI. #: Significant differences between the normal group and the non-lesion side of PD group by ANCOVA after controlling for age, sex, and BMI. Abbreviations: PD: Parkinson’s disease; LS: lesion side; NLS: non-lesion side.

**Table 3:** Comparison between fat fraction and cross-sectional areas (mm^3^) of the lesion and non-lesion side in PD patients and normal controls.

**Fat Fraction (%)**	Normal Controls	PD_LS_	*p*	PD_NLS_	*p*
Psoas muscles	9.16 ± 2.01	11.77 ± 4.71	0.003 *	11.08 ± 4.01 ^#^	0.038 *
Anterior compartment of thigh	8.65 ± 2.83	11.17 ± 2.91	0.002 *	10.87 ± 2.84 ^#^	0.016 *
Medial compartment of thigh	8.18 ± 1.77	11.59 ± 2.99	0.000 *	10.62 ± 4.10 ^#^	0.007 *
Posterior compartment of thigh	12.27 ± 3.46	16.83 ± 5.31	0.000 *	17.26 ± 6.05 ^#^	0.000 *
Total thigh	13.51 ± 3.19	16.05 ± 3.84	0.008 *	16.27 ± 4.93 ^#^	0.026 *
**Cross-Sectional area (mm^3^)**	Normal Controls	PD_LS_	*p*	PD_NLS_	*p*
Psoas muscles	806.80 ± 276.63	844.49 ± 275.11	0.637	901.71 ± 284.93	0.584
Anterior compartment of thigh	5267.33 ± 960.91	5151.86 ± 1094.41	0.027 *	5601.82 ± 1140.72	0.945
Medial compartment of thigh	2872.52 ± 626.79	2628.61 ± 660.64	0.031 *	2590.92 ± 501.83	0.017 *
Posterior compartment of thigh	1511.22 ± 399.79	1800.75 ± 394.60	0.042 *	1919.07 ± 513.87	0.007 *
Total thigh	10,809.89 ± 1,814.65	10,590.24 ± 1,771.51	0.004 *	11,200.29 ± 1,956.08	0.272

Fat fraction and cross-sectional data were compared by analysis of covariance (ANCOVA) after controlling for age, sex and BMI. Data are presented as mean ± standard deviation; * *p* < 0.05. Abbreviations: PD: Parkinson’s disease; LS: lesion side; NLS: non-lesion side.

**Table 4:** Comparison of fat fraction and cross-sectional areas (mm^3^) in female and male groups.

**Female/Male groups**	PD, female, n = 20	NC, female, n = 16	*p*	PD, male, n = 5	NC, male, n = 4	*p*
Age	63.80 ± 5.46	62.38 ± 3.63	0.377	62.8 ± 6.42	65.50 ± 5.45	0.525
BMI	25.27 ± 3.65	22.76 ± 3.72	0.053	24.42 ± 4.78	24.69 ± 2.20	0.920
**Fat Fraction (%)**			*p*			*p*
Psoas muscles	11.89 ± 3.75	9.14 ± 2.28	0.014 *	9.90 ± 2.53	9.25 ± 0.96	0.645
Anterior compartment of thigh	11.55 ± 2.82	9.16 ± 2.79	0.016 *	8.64 ± 1.11	6.61 ± 2.15	0.108
Medial compartment of thigh	12.86 ± 4.48	8.61 ± 1.71	0.001 *	9.48 ± 2.50	6.46 ± 0.31	0.053
Posterior compartment of thigh	17.21 ± 5.37	12.87 ± 3.64	0.009 *	12.63 ± 3.02	9.88 ± 0.21	0.119
Total thigh	17.66 ± 4.00	14.44 ± 2.52	0.008 *	11.59 ± 1.24	9.88 ± 0.31	0.263
**Cross-Sectional Area (mm^3^)**			*p*			*p*
Psoas muscles	770.25 ± 150.54	700.47 ± 174.58	0.207	1255.91 ± 304.67	1232.11 ± 175.48	0.894
Anterior compartment of thigh	4940.69 ± 864.43	4931.28 ± 652.32	0.971	6678.60 ± 1040.19	6611.50 ± 842.03	0.920
Medial compartment of thigh	2469.79 ± 449.27	2702.78 ± 584.89	0.185	3246.75 ± 541.33	3551.56 ± 95.60	0.309
Posterior compartment of thigh	1784.91 ± 391.55	1383.51 ± 251.04	0.001 *	2021.60 ± 553.58	2022.05 ± 512.19	0.999
Total thigh	10,290.29 ± 1410.12	10,200.82 ± 1282.27	0.845	12,744.46 ± 2217.25	13,246.16 ± 1655.87	0.719
